# Experiences of critical care nurses infected with COVID-19 in a Saudi Academic Hospital

**DOI:** 10.4102/curationis.v48i1.2735

**Published:** 2025-05-07

**Authors:** Donald Mazibu, Charlene Downing, Richard Rasesemola

**Affiliations:** 1 Department of Nursing, Faculty of Health Sciences, University of Johannesburg, Johannesburg, South Africa

**Keywords:** caring, CC nurses, experience, mental health, resilience, Saudi Arabia, social isolation or quarantine

## Abstract

**Background:**

Coronavirus disease 2019 (COVID-19) has severely impacted nursing services, with thousands of ICU nurses infected with potentially fatal respiratory infections. Saudi Arabian studies suggest psychological implications, with loneliness and social isolation linked to higher depression and anxiety levels. Pandemic preparedness should focus on workforce protection and mental well-being promotion.

**Objectives:**

This study aimed to understand the experiences of critical care nurses in Saudi Arabia who contracted COVID-19, with the goal of formulating recommendations to empower them, increase resilience, and help the profession thrive post-pandemic.

**Method:**

This study used an exploratory, descriptive, and contextual approach, conducting unstructured interviews with 11 Critical Care Unit (CCU) Registered Nurse (RN) who contracted COVID-19, and analysing the data using Colaizzi’s seven-step method.

**Results:**

Three themes emerged from the data: (1) physical isolation led to loneliness and psychological separation, lacking emotional support and freedom of choice; (2) reintegration into the work environment increased stress and loneliness and (3) Coronavirus disease 2019 exposure provided essential insight into patient care and increased awareness of adaptive skills. These themes highlight the challenges participants faced during the pandemic.

**Conclusion:**

This research study indicated the experiences of ICU nurses who contracted COVID-19, revealing feelings of loneliness, depression and stress. Despite reintegration into their work environment, these feelings persist.

**Contribution:**

The study indicates that nurses in social isolation or quarantine also require open communication, social interaction, personal autonomy and emotional support to boost their resilience and mental health, and to increase their job satisfaction.

## Introduction

### Background

Coronavirus disease 2019 (COVID-19) was discovered in Wuhan City in December 2019. The disease spreads through direct contact, respiratory droplets and fomite (Jebril 2020; Said & El-Shafei 2021; Singhal 2020). Fever, coughing, sore throat, headaches, fatigue, myalgia and shortness of breath are all common clinical symptoms (Jebril 2020; Singhal 2020; World Health Organization [WHO] 2020). With thousands of people infected with potentially fatal respiratory infections, the pandemic has put a significant strain on nursing services (Algaissi et al. 2020; Jebril 2020). The recommendation is for the intensive care unit (ICU) community to prepare for a surge of patients by optimising workflows for rapid diagnosis, isolation, clinical management and infection prevention (Phua et al. 2020). On 02 March 2020, the Kingdom of Saudi Arabia announced the first case of COVID-19 and announced free medical care for all patients in public and private healthcare institutions (Jebril 2020; Meo 2020).

In April 2020, the Saudi Arabian Ministry of Health (MOH) reported an increase in COVID-19 cases, prompting strict measures such as restricting outdoor activities, suspending schools, limiting social contact, prohibiting mosque prayers and imposing a nationwide lockdown (Alahdal, Basingab & Alotaibi 2020). Because of the increased demand for ICU beds, hospitals were forced to convert general wards into ICUs (Alahdal et al. 2020). The Saudi Safety Patient Centre (SPSC) advocated for improved critical care and nursing staffing models, as well as reducing elective services and surgeries to free up space for critical care services. The SPSC also emphasised the importance of teaching, cross-training and ensuring that healthcare workers are properly trained (SPSC 2020). In light of previous middle east respiratory syndrome (MERS) epidemics, Saudi Arabia’s public health system, infection control policies and measures were better prepared for the COVID-19 pandemic (Algaissi et al. 2020). The hospital’s critical care nursing administration unit used a ‘pod’ allocation system, with one pod consisting of one ICU RN and one non-ICU RN caring for two ICU patients with careful donning and doffing of personal protective equipment (PPE) as a key defence against COVID-19 infection.

In assessing RNs readiness to care for COVID-19 patients, 8.6% of RNs reported feeling ‘prepared’, while 32.0% indicated they were ‘somewhat prepared’. Notably, nearly half of the RNs (45.2%) expressed uncertainty regarding their preparedness (Labrague & De Los Santos 2020). Registered nurses experienced high levels of anxiety and stress during the COVID-19 outbreak, with burnout scores ranging from 31.0% to 54.3% (Tayyib & Alsolami 2020). Emotional exhaustion, depersonalisation, decreased personal accomplishment, moral distress and high levels of depression, stress, anxiety, anger, fear, insomnia and post-traumatic stress disorder are all symptoms of burnout (Heath, Sommerfield & Von Ungern-Sternberg 2020; Shaukat, Ali & Razzak 2020; Sultana et al. 2020; Vranceanu 2019). Understanding the consequences of burnout is critical for personal well-being and patient care (Heath et al. 2020). The high stress and fear of COVID-19 infection among RNs has a negative impact on care quality and safe practise (Sultana et al. 2020).

During a pandemic, the WHO stresses the significance of managing mental and psychosocial well-being (WHO 2020). Having a happy life, upholding moral principles, cultivating strong social bonds, employing the right life skills to overcome obstacles and having access to high-quality services are all components of well-being (Inter-Agency Network for Education in Emergencies [INEE] 2014). Reducing health consequences can be achieved by putting in place appropriate PPE training, stringent infection control procedures, shorter shifts and mental health support services (Shaukat et al. 2020). Intensive care unit RNs’ psychological health can be enhanced and anxiety levels can be reduced with the implementation of a documented outbreak management plan. One tactic for encouraging and keeping healthcare staff is ‘risk allowance’ (Chersich et al. 2020). Increasing the positive emotions and psychological resilience of ICU RN is essential during pandemics, as it lowers burnout and compassion fatigue and raises occupational satisfaction, care quality and patient satisfaction (Ata, Yılmaz & Bayrak 2020). Studies show that nurses with higher resilience scores have reduced COVID-19 anxiety levels (Labrague & De Los Santos 2020).

Studies suggest enhancing health education through online platforms and addressing social fear related to COVID-19 (Serafini et al. 2020). This includes the importance of addressing stigma and discrimination, which can exacerbate uncertainty during a crisis. Hospital policies should manage health emergencies effectively, while healthcare workers need adequate protective facilities (Serafini et al. 2020). Routine mental status check-ups are suggested to ensure physical needs are met (AlAteeq et al. 2020). People fear the future, and nurses who served during the pandemic should be appreciated for their sacrifices and more research should be dedicated to their productivity, focus and livelihood (Buheji & Buhaid 2020; Horesh & Brown 2020). The hospital under study had established mental health support systems, and notifications regarding these services were disseminated via email. However, the majority of RNs were unaware of these services. In addition, there was a reported stigma associated with seeking mental health consultation among the RNs.

Access to mental health support and high-quality qualitative research are needed to better understand RNs’ experiences, needs and preferences post-pandemic.

### Research aim

The aim of this study was to gain an understanding of the lived experiences of critical care RNs who contracted COVID-19 in an academic hospital in Saudi Arabia, with the intension of formulating recommendations to facilitate their empowerment, maximise resilience and help the profession thrive post-pandemic.

## Research designs and methods

### Research design

The author conducted a qualitative article using an exploratory, descriptive and contextual approach to better understand the significance of participant’s experiences (Polit & Beck 2021). The author employed constructivism and naturalism to investigate various interpretations of reality, resulting in a better understanding of the participants’ ideas and feelings (Ahmed 2008). This article investigates the lived experiences of critical care RNs who contracted COVID-19 while working in a Saudi academic hospital.

### Research setting

This study was conducted in Saudi Arabia among RNs working at King Fahad Medical City (KFMC). The facility has 537 ICU RNs from 12 different countries. The majority (88.45%, *n* = 475) of RNs were from Asian countries.

### Population

The target population in this study included RNs working in an ICU at King Fahad Medical City. Participants were chosen based on specific inclusion criteria for the article, which used a descriptive phenomenological research method (Creswell & Poth 2018). Population in this study include 537 RNs working in an ICU. The target population included those ICU RNs who contracted COVID-19 while working in an ICU.

### Sampling

Purposive sampling was used to recruit participants according to the inclusion criteria. The inclusion criteria for this study comprised ICU RNs who contracted COVID-19, who have been working in an ICU at the time of data collection and have been exposed to critically ill COVID-19 patients.

### Data collection

After obtaining ethical clearance and permission from the Faculty of Health Sciences, the Research Ethics Committee, the Higher Degrees Committee of the Faculty of Health Sciences at the University of Johannesburg, and the KFMC hospital’s ethics committee, the researcher sent a request to conduct the study to the hospital’s critical care nursing director. The executive director of nursing granted permission to conduct the study after the nursing research committee reviewed the proposal. The researcher recruited the potential participants by distributing posters and pamphlets around the hospital inviting ICU RNs to participate in the study. For those who showed interest, the researcher held an information session where all prospective participants had the opportunity to receive, read and interpret the information letter, which explained why the research was being conducted and what participation involved. Participants who were willing to participate signed a consent form. Appointments for those who signed consent to participate in the study were made. Data collection was conducted by the researcher through interviews and field notes between 25 June 2022 and 21 March 2023. Each interview lasted between 47 min and 60 min, was audio-recorded and subsequently transcribed by the researcher. Individual unstructured interviews were conducted at a location and time convenient for both the participants and the interviewer; it was a private space with few interruptions. The researcher posed an open-ended question to the research participants: ‘Tell me about your experiences after you contracted COVID-19?’. ‘How did you experience the transition back to work after being discharged?’. In exploring individuals’ lived experiences with COVID-19, it is essential to understand both their immediate and long-term challenges. The central question, ‘Tell me about your experiences after contracting COVID-19?’ seeks to capture the physical, emotional and psychological impacts of the illness. The follow-up question, ‘How did you experience the transition back to work after being discharged?’ provides deeper insight into the recovery process and reintegration into daily life. Examining these aspects, the study aims to offer a comprehensive understanding of the post-COVID experience, particularly in relation to personal well-being and professional responsibilities. The researcher also took field notes that were used during data analysis.

### Data analysis

Colaizzi’s seven-step method was used for data analysis. The process involved organising, coding and condensing collected data into themes, which were then presented through figures, tables or discussions, using both field notes and recorded interviews (Creswell & Poth 2018). The researcher first identified key concepts, categorised the data into specific codes and then organised these codes into nine distinct categories. The researcher and the independent coder shared similar perspectives on the identified themes and ultimately reached a consensus on the final number of themes. These categories were further refined and consolidated into three overarching themes, ensuring a coherent and structured analysis.

### Ethical consideration

Ethical approval and permission to conduct this study were obtained from the Faculty of Health Science Research Ethics Committee (REC-1092-2021), the Higher Degrees Committee of the Faculty of Health Sciences at the University of Johannesburg (HDC-01-38-2021) and the King Fahad Medical City (KFMC) Hospital Ethics Committee (IRB log number: 21-293).

The study received ethical approval from relevant committees and nursing leadership, with ICU RNs invited to participate through informational materials and sessions. Participants signed an informed consent reflecting their willingness to participate in the study. The researcher ensured participants privacy and confidentiality by creating a password for a folder containing participants’ data to limit access to the authorised personnel. An independent coder also had to sign a confidentiality agreement.

## Results

Findings will be discussed in this section including demographic data of the participants and the themes that emerged from data analysis.

### Demographic data

Participants’ demographic data, including their age in years, their qualifications, gender, ethnicity, citizenship and ICU experience are presented in Table 1.

**TABLE 1:** Participants’ demographic information.

Participant	Age in years	Qualifications	Gender	Ethnicity	Citizenship	ICU experience in years
1	34	Bachelor of Science in Nursing	Female	Asian	Indian	13
2	29	Master of Nursing Science	Female	Asian	Filipino	13
3	39	Diploma in Nursing Science	Female	Asian	Indian	17
4	28	Bachelor of Science in Nursing	Female	Asian	Indian	6
5	29	Bachelor of Science in Nursing	Female	Asian	Saudi Arabian	6
6	29	Bachelor of Science in Nursing	Female	Asian	Filipino	7
7	33	Bachelor of Science in Nursing	Female	Asian	Philippines	11
8	23	Bachelor of Science in Nursing	Female	Asian	Saudi Arabian	2
9	29	Bachelor of Science in Nursing	Female	Asian	Filipino	5
10	33	Bachelor of Science in Nursing	Female	Asian	Filipino	13
11	37	Bachelor of Science in Nursing	Female	Asian	Indian	12

*Source*: Mazibu, D., 2023, ‘Experiences of critical care registered nurses who contracted Covid-19 in an academic hospital in Saudi Arabia’, Unpublished Master’s Degree, University of Johannesburg, Johannesburg.

ICU, intensive care unit.

The final sample size in the study was determined by the point at which sufficient and relevant data had been collected, and no new information, codes or themes were emerging from the data (Johnson, Adkins & Chauvin 2020). Eleven participants were interviewed, their age ranged from 23 to 37 years. Participants were all female, all with an Asian Ethnic background, and four of the participants were from India, five were from the Philippines and two were from Saudi Arabia. Participants’ experience in ICU varied greatly, six of the participants had experience of more than 10 years with one having experience of 17 years, while five had experience of less than 10 years.

### Themes and categories

Three themes and nine related categories emerged from the data and are presented in Table 2.

**TABLE 2:** Themes and categories.

Themes	Categories
1. Physical isolation led to loneliness, a psychological separation lacking emotional support and freedom of choice	1.1 The diagnosis and isolation process. 1.2 Loneliness kicked in, causing immense psychological distress and suffering. 1.3 Participants lacked emotional support and freedom of choice.
2. Reintegration into the work environment increased stress and loneliness because of watchfulness	2.1 Participants made every effort not to infect anyone at work – not their co-workers or patients – and not to be re-infected. 2.2 Participants noticed that staff members were avoiding them to protect themselves, and although they could understand this response, it was still hurtful. 2.3 There was a shortage of staff as well as PPE, which added to their stress about contagion.
3. COVID-19 exposure provided essential insight into patient care and increased awareness of adaptive skills	3.1 Participants gained much-needed insight into pandemics. 3.2 Participants became aware of their own levels of compassion and care for their patients. 3.3 Participants realised that they developed adaptation skills.

*Source*: Mazibu, D., 2023, ‘Experiences of critical care registered nurses who contracted Covid-19 in an academic hospital in Saudi Arabia’, Unpublished Master’s Degree, University of Johannesburg, Johannesburg.

PPE, personal protective equipment; COVID-19, coronavirus disease 2019.

The participants were diagnosed with COVID-19, which resulted in a difficult period of physical isolation that had a significant impact on their mental and physical well-being. Participants reported feelings of loneliness and withdrawal upon returning to work. The participants also had to adjust to new roles and departments, which included shifts between paediatric and adult ICUs. The themes and categories will be discussed in the section with supporting quotations from the participants.

### Theme 1: Physical isolation led to loneliness, a psychological separation lacking emotional support and freedom of choice

Participants reported feeling isolated and distressed because of the lack of face-to-face interaction, physical touch and participation in activities. They were not allowed to leave the quarantine area, and food was delivered outside the door. This isolation led to a lack of social interaction, emotional support and freedom to make daily decisions, exacerbated feelings of isolation, anxiety and depression. The author used participants’ verbatim accounts to support this discussion.

#### Category 1.1: The diagnosis and isolation process

The COVID-19 diagnosis and isolation process involve multiple steps, including testing individuals with symptoms using a polymerase chain reaction (PCR) swab at a healthcare facility to detect the virus in their nose or throat:

‘I went to the hostel I was sleeping when I wake up from the night shift, I got like something in my body, it’s my body, too much pain, and I started to cough like mild cough then I noted I took the paracetamol then after that I’m about to come to the duty. I thought that there is something wrong with me. It’s like my voice is going down and there is severe throat, sore throat so I just informed my unit that I’m not going to come, then I just went to the hospital to test for COVID.’ (Participant 1)

‘It felt like I’m having and coughing already and that’s the peak where, here in the hospital is very strict. If you’re having cough you need to do the swab at that time. So, I’ve decided to go in our clinic and do the swab.’ (Participant 7)

#### Category 1.2: Loneliness kicked in, causing immense psychological distress and suffering

Participants experienced severe psychological distress and loneliness because of a sudden sense of being disconnected from their daily lives, leading to fear, depression and a lack of social interaction:

‘I mean, you cannot go out outside of the room and you have to be in the room. You cannot conduct with any other people by I mean you’re a real really you are isolated from the from the social life. I mean, you cannot go outside, and you will be alone, you’ll feel alone.’ (Participant 4)

‘But on the 4th day of the quarantine itself, so, so, so, so what I call this? You feel the isolation already, like you cannot do anything. You’re just in the floor, like four walls and then you want to free yourself. You want to do something like this, but you cannot do because you’re isolated.’ (Participant 8)

#### Category 1.3: Participants lacked emotional support and freedom of choice

Participants reported having limited access to emotional support and psychological counselling, with the majority lacking contact information for emotional support:

‘Actually, I was symptomatic at that time, I have like sore throat and, and I have fever on and off. So, I decided to go to uh, swabbing at that time in the, in our OPD and then I have not been contacted actually where I should. Yeah no, I actually went to the ER at that time because I’m not really feeling well.’ (Participant 8)

‘Yeah, one of the things there is no socialization. Like I am in a jail, you know. And I’m calling my family and talking to them. That is all okay, but I’m. I’m not seeing anyone outside and my friends they are also calling, but I’m not seeing them. I’m not going outside. For me, it’s difficult for me to sit inside that will not work for seven days. So, it’s quite somehow depressing.’ (Participant 11)

### Theme 2: Reintegration into the work environment increased stress and loneliness because of watchfulness

The lack of informal interactions and gatherings caused participants to feel disconnected from their colleagues, which was exacerbated by ongoing concerns about the virus and personal losses. The emotional strain and increased stress of the participants were exacerbated by frequent changes in rules, workplace policies and organisational structures.

#### Category 2.1: Participants made every effort not to infect anyone at work – not their co-workers or patients – and not to be re-infected themselves

Participants demonstrated a strong commitment to preventing infection spread and protecting themselves and others in their workplace, learning from their experience with COVID-19 and demonstrated a caring behaviour by advocating for patients and protecting colleagues from infection:

‘But we need to do some of the precautions so that we need so we can avoid the spread of the virus. And also, for the sake of the patients, because the patient is much more weaker than us because they’re sick. So yeah.’ (Participant 7)

‘Every time I’m coming to duty, I think oh […] you need to have this space you need. You cannot infect your colleague because they also have family what if something happened to them.’ (Participant 6)

#### Category 2.2: Participants noticed that staff members were avoiding them to protect themselves, and although they could understand this response, it was still hurtful

In a difficult situation, participants experienced conflicting emotions while watching colleagues create social distance to protect themselves from potential infection. Despite their understanding of the emotional impact of this self-preservation action, the emotional toll on them was undeniable:

‘Even if you’re negative, you have to make their social distance with everyone. And maybe they will, they will look at you like you’re infected with COVID-19. So, you have to face all those things to fix your mind to feel that way.’ (Participant 4)

‘I don’t think that they, I don’t know if they care, if, uh, if they care that I’m not going with them or if they want me to talk with them. But I thought like its might just be not to join them for now, since I just came back from this, uh, viral infection.’ (Participant 2)

#### Category 2.3: There was a shortage of staff as well as personal protective equipment, which added to their stress about contagion

The practice of ICU RNs reusing gowns and masks because of lack of PPE also raised concerns about their health and safety, despite the necessity of this practice, which can cause discomfort and anxiety:

‘It’s also scary because you will feel guilty if you’re going to spread the virus to your friend, although we cannot avoid that sometimes.’ (Participant 7)

‘Well, the hospital has been, uh, very active in updating the staff about the protocol. Although it keeps on changing. There are always, this hospital always keeps on messaging us in our email and officially email of the hospital.’ (Participant 2)

### Theme 3: Coronavirus disease 2019 exposure provided essential insight into patient care and increased awareness of adaptive skills

The COVID-19 pandemic has presented special challenges for healthcare workers. They have firsthand experience managing and treating patients during the pandemic. Through improved knowledge of preventive measures such as immunisation, handwashing and mask use, participants were more likely to follow health advice and stop the spread of viruses. Participants also gained knowledge about mental health concerns and the significance of seeking resources and assistance.

#### Category 3.1: Participants gained much-needed insight into pandemics

The COVID-19 pandemic has resulted in a significant educational initiative, providing participants with critical measures and protocols to combat the health crisis, fostering confidence and readiness for future pandemics, and providing them with valuable knowledge:

‘Well, the hospital has been, uh, very active in updating the staff about the protocol. Although it keeps on changing. There are always, this hospital always keeps on messaging us in our email and officially email of the hospital. Uh, and also, um, some of our in-services, although it was online, I cannot actually remember if there. If online in services was already happening that time.’ (Participant 2)

‘So that’s the positive thing of having this kind of disease during that time. So that in the future you know already what will happen, what management or what will, what you’re gonna do for you not to have this kind of infection already.’ (Participant 11)

‘I felt like I developed some resistance with the COVID-19, so it didn’t bother me handling patients with active, uh, active, doing the active phase of the COVID-19. So, I don’t know, I, it felt normal with me. It felt like I don’t have any fear in having them anymore because even though they’re COVID-19 active condition. Uh, it did not border me actually, it did not boarder me.’ (Participant 2)

#### Category 3.2: Participants became aware of their own levels of compassion and care for their patients

Participants felt empathy for patients whose families could not visit them in the paediatric ward, experiencing emotional pain because of the lack of parental support during trying times:

‘Uhm, that time actually it’s very stressful also I think for the families, because they are really not allowed to see all their uhm, child especially if it’s admitted in the critical ICU’s. I mean, visiting hour is already prohibited at that time.’ (Participant 8)

‘I also feel sad for them because sometimes they’re crying and then even though we’re trying to consulate them, still they’re crying. They’re not, they don’t feel relaxed about us because they don’t know us. They don’t know us. So, I also feel sad for them because they’re searching for the for, for their parents, so yah.’ (Participant 7)

#### Category 3.3: Participants realised that they developed adaptation skills

During the pandemic, participants faced and conquered numerous challenges, enhancing their appreciation for their loved ones and the time spent together. The pandemic strengthened participants’ spirituality, gratitude and resilience, while also improving their understanding of pandemics, compassion for patients and critical adaptation skills for overcoming adversity:

‘Since uh, new awareness, new knowledge for the new knowledge for this kind of infection and how can you prevent it in the future if it will happen again. So, because it’s not always uh what is this? in such an experience, so you know how to prevent it already. The next time it will happen, you will know already the measures.’ (Participant 11)

‘I am entering patient before with N95 then after also I am dealing with patients with my N95. And yeah, and maybe let me thing still I am fixing the N95 more tightly because they became big more big.’ (Participant 9)

‘So, you have to talk to the patient and when you will go with these all PPEs, they cannot see your face okay, only by voice and you don’t know their language. Okay, same, like, there are some there, at least if you know their language, at least they can, we can communicate with them so they will be happy. But for us, it’s very difficult situation.’ (Participant 3)

‘This is what I’m thinking as the person, okay. it’s like everyone in my life is important for me. I need to be close with them, I don’t know what will happen to us.’ (Participant 6)

‘So, you’re exposed at this time, this might be helpful in the future that you might be encountering this kind of patient and you already have the idea, which is very helpful, so.’ (Participant 8)

## Discussion

Physical isolation led to profound loneliness, depression, anxiety and loneliness because of the lack of social interaction (Joseph et al. 2022). The isolation prevented participants from participating in normal social activities and interactions, and there was a significant lack of emotional support. The lack of leadership support can lead to emotional distress and burnout (Rangachari & Woods 2020).

The limited freedom to make decisions in daily life exacerbates feelings of isolation and discomfort. The participants were aware of the necessity for isolation and actively enforced similar restrictions on their patients. However, despite their understanding of the importance of isolation, the experience they imposed on their patients was not perceived as positive, as it often resulted in a lack of social contact and its associated negative impacts. Participants felt disconnected from their usual lives, missing their families and longing for loved ones, which exacerbated their emotional distress. While social restrictions are required to prevent the spread of COVID-19, it is critical to remember that physical distancing does not equal social disconnection (Hwang et al. 2020).

Theme one addresses the emotional and psychological challenges faced by critical care RNs during physical isolation. The person comprises a unity of mind, body, spirit and nature within an evolving worldview that connects all. In this state, a critical care RN is disconnected from the environment and its components, including friends, family and the community. The participants’ feelings, energy, comfort and capacity to carry out their daily tasks are out of balance because of their disconnection from the environment.

The recommendations in this article emphasise the importance of social interaction, emotional support and personal autonomy in maintaining mental well-being, as well as the devastating impact of isolation on an individual’s mental health. Coping mechanisms, psychological resilience and social support are essential for maintaining ICU RNs’ mental and psychological well-being during disease outbreaks such as the COVID-19 pandemic (Labrague 2021). Positive coping mechanism, such as mindfulness, physical exercise, hobbies and professional counselling, have been shown to improve mental health (Sultana et al. 2020). The importance of training in self-efficacy and coping skills to help ICU RNs manage the increased pressures of the COVID-19 pandemic (Labrague 2021) were highlighted. Psychological resilience, fostered through a positive outlook, adaptability, emotional regulation and reflection on past successes, along with strong social support from colleagues, family and managers, is essential for maintaining ICU RNs’ well-being during disease outbreaks (Labrague & De Los Santos 2020).

Increased autonomy in unit operations decisions can improve job satisfaction and overall well-being (Inter-Agency Standing Committee [IASC] 2020).

Participants returned to their workplace after contracting COVID-19, observing their colleagues taking precautions to prevent contracting the virus. These measures included face masks, social distancing and extended hygiene practices. However, these measures increased stress for the participants, leading to feelings of loneliness and sadness. The stigma linked to COVID-19 can cause social exclusion among healthcare workers, who may face altered anxiety levels because of fear of contracting the infection, spreading the disease to loved ones, lack of information and scarcity of PPE (IASC 2020). The UNAIDS (2020) highlighted the fact that the fear of stigma during a pandemic can deter healthcare workers, including RNs, from adhering to preventive measures, delay timely testing and undermine compliance with treatment protocols. Furthermore, the IFRC (2023) emphasised the fact that stigma and misconceptions surrounding mental health often led individuals to hesitate in seeking support and treatment, resulting in self-isolation. The International Council of Nurses (ICN) (2023) highlights the fact that factors such as pathogen exposure, long working hours, psychological distress, fatigue, occupational burnout, stigma and physical and psychological violence significantly harm nurses’ health and well-being. Addressing stigma, in particular, is crucial to mitigating these negative impacts and improving overall outcomes for nursing professionals.

Theme two suggests that even when nurses returned to their workplaces, the ongoing need for virus prevention measures added a layer of stress and emotional strain, potentially perpetuating feelings of loneliness and sadness. Critical care RNs experienced extended feelings of loneliness because of a lack of knowledge about the virus’s transmission and fear of infecting others. To maintain their health, critical care RNs should receive appropriate PPE, clear communication and education about the virus’s transmission and its effects on health. UNAIDS (2020) recommends training and information for loved ones and carers of COVID-19 patients to reduce the risk of transmission, address misconceptions and reduce stigma associated with caring for a person with COVID-19.

The participants learned about COVID-19 protocols and measures, feeling immune to the virus and believing the vaccines were effective. This experience prepared them for future pandemics and helped them become more aware of their compassion and care for their patients. The pandemic experience also made participants more aware of their adaptive abilities, learning new skills to deal with the challenges the pandemic created.

Theme 3 indicates that critical care RNs are likely better prepared to face future challenges because of their knowledge and self-awareness. Resilience derived from self-efficacy and necessary coping skills can assist nurses in dealing with difficult situations (Yu et al. 2019). Personal growth and increased capacity to deal with future challenges are often the result of resilience.

**FIGURE 1:**
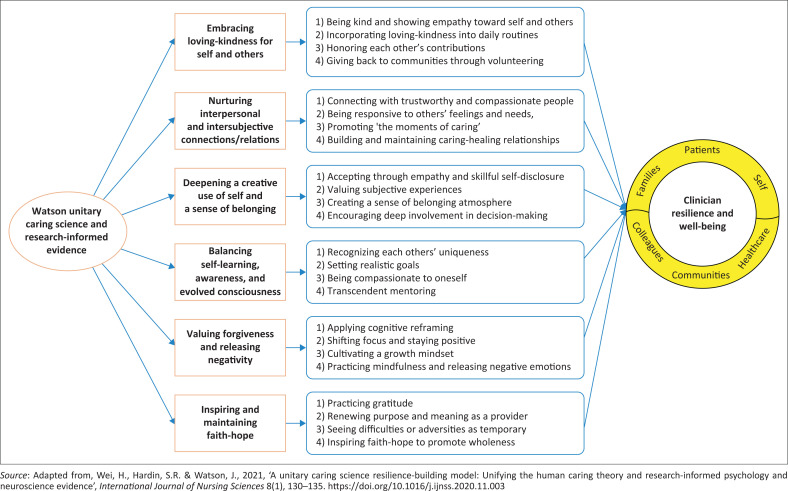
Unitary caring science resilience-building model.

The health facilities must implement the six-strategy resilience-building model, which is based on unitary caring science and evidence informed by research, to guarantee nurses’ resilience in the face of difficult circumstances (Wei, Hardin & Watson 2021) (see Figure 1). Baluszek, Brønnick and Wiig (2023) emphasised that leaders should cultivate a supportive work environment by promoting a positive workplace culture, which can reduce staff stress, enhance employees’ self-efficacy and strengthen their psychological well-being. Leaders must monitor and address the mental health of RNs, as this is essential for enhancing psychological resilience and reducing burnout (Van den Broek, Galffy & De Vroege 2023). Wei et al. (2021) further elaborated that a healthcare facility is considered resilient if its employees receive support at all three levels (individual, team and healthcare facility) of foresight, coping and recovery.

### Strength and limitations

The article was conducted in ICUs with 144 beds and a workforce comprising 531 critical care RNs. Eleven RNs voluntarily participated, representing a significant portion of the workforce. Unstructured interviews were used to explore the lived experiences of COVID-19 patients. Data saturation was achieved after the ninth interview, indicating a comprehensive understanding of the participants’ experiences. Interviews were conducted in participants’ settings, creating a comfortable environment for open sharing. The author incorporated current research articles to support the discussion, enhancing the article’s credibility. The findings and recommendations are expected to contribute valuable insights to the nursing research body of knowledge.

A major limitation of this study is its reliance on one country and institution, limiting its generalisability, and its exclusive sample from the same institution, potentially limiting diversity. The article’s female participants, reflecting the gender distribution in the critical care department, may impact the comprehensiveness of its insights. In addition, the article’s focus on the lived experiences of critical care RNs who contracted COVID-19, excluding perspectives from other nursing specialties or departments, may not provide a holistic view of the challenges faced by the entire nursing community. Further samples from different institutions and a more diverse range of nursing specialties could enhance the research’s scope and depth.

### Recommendations

Leaders should prioritise the development of programmes and initiatives intended at fostering a supportive work environment and promoting a positive workplace culture. This includes implementing regular mental health check-ins, providing access to resources such as counselling and stress management workshops and offering training to enhance self-efficacy and emotional resilience among RNs. Open communication, social interaction, personal autonomy and emotional support for nurses experiencing social isolation or quarantine should be prioritised. This will promote mental health and resilience, boosting employee engagement and job satisfaction. Empowering nurses with stress management and coping skills can also enhance resilience.

## Conclusion

The article revealed the experiences of critical care RNs who contracted COVID-19, revealing feelings of loneliness, depression and stress. Despite reintegration into their work environment, these feelings persisted. The research suggests that mental health support and resilience training are crucial for nurses experiencing emotional and psychological stress.
